# We are also interested in how fathers feel: a qualitative exploration of child health center nurses’ recognition of postnatal depression in fathers

**DOI:** 10.1186/s12884-015-0726-6

**Published:** 2015-11-09

**Authors:** Kina Hammarlund, Emilie Andersson, Hanna Tenenbaum, Annelie J. Sundler

**Affiliations:** School of Health and Education, University of Skövde, Box 408, Skövde, SE–541 28 Sweden; Home healthcare, Falköping Municipality, Falköping, Sweden; Primary healthcare, Vara, Sweden; Faculty of Caring Science, Work Life and Social Welfare, University of Borås, Borås, Sweden

**Keywords:** Father, Interview, Depression, Nursing, Qualitative

## Abstract

**Background:**

To become a parent is an emotionally life-changing experience. Paternal depression during the postnatal period has been associated with emotional and behavioral problems in children. The condition has predominantly been related to mothers, and the recognition of paternal postnatal depression (PND) has been paid less attention to. PND in fathers may be difficult to detect. However, nurses in pediatric services meet a lot of fathers and are in a position to detect a father who is suffering from PND. Therefore, the aim of this study was (a) to explore Child Health Center nurses’ experiences of observing depression in fathers during the postnatal period; and (b) to explore hindrances of observing these fathers.

**Methods:**

A qualitative descriptive study was conducted. Ten nurses were interviewed in 2014. A thematic data analysis was performed and data were analyzed for meaning.

**Results:**

Paternal PND was experienced as being vague and difficult to detect. Experiences of fathers with such problems were limited, and it was hard to grasp the health status of the fathers, something which was further complicated when routines were lacking or when gender attitudes influenced the daily work of the nurses.

**Conclusion:**

This study contributes to an increased awareness of hindrances to the recognition of PND in fathers. The importance to detect all signals of paternal health status in fathers suffering from PND needs to be acknowledged. Overall, more attention needs to be paid to PND in fathers where a part of the solution for this is that they are screened just like the mothers.

## Background

To become a parent is one of the most emotionally life-changing experiences in a person’s life. During this first period after the baby is born, some parents suffer from postnatal depression (PND) [1]. PND includes symptoms such as a lower mood, feelings of guilt and problems with concentration, as well as feelings of losing control of one’s life and not being good enough as a parent [2]. Stressful life events have been found to be associated with maternal depression during both pregnancy and the whole first postnatal year. This was not the case for depression in fathers. Fathers who had a history of depression were found to be more vulnerable to PND. They were also more likely to be depressed if their partner had been depressed during pregnancy and in the first three months after birth [3].

According to Bowlby’s theory [4] of the attachment process, a new-born baby has an instinctive need to seek closeness and contact by smiling or crying. If the parents respond to these signals, the baby will develop a secure base and attach to the parents [4]. This early interaction can be disrupted if a parent is afflicted with depression, which may cause future behavioral problems and affect cognitive functions [5–7]. Paternal depression during the postnatal period has been associated with emotional and behavioral problems in children [5] later in childhood, children of depressed fathers remain at increased risk [8]. According to [9], boys seem to be more vulnerable than girls to the effects of paternal depression, especially during early development.

In Sweden, pediatric care is offered to all children from birth to six years of age, the health service is free of charge and voluntary. The basis of this service is to support health and prevent ill-health in children [10–12]. At a Child Health Center (CHC), a nurse who specializes in infants meets the parents and arranges a visit in their home when the baby was newly born. The most common complication of childbearing is PND. A widely used instrument for screening for PND in mothers in the year following the birth of a child is the Edinburgh PND Scale (EPDS) [13, 14]. Screening for depression in the postnatal period in fathers has received relatively little research attention and routines for screening fathers are less well-developed in practice [1]. If a parent is suffering from PND, it may affect interactions with not only the baby but the entire family. Earlier research has focused on this type of depression concerning mothers, but fathers can also suffer from PND [1, 5, 8, 9]. PND in fathers may be difficult to detect and nurses in pediatric services have a key position to meet and give support to these fathers [7]. To be able to do that, more research is needed from the point of view of the CHC nurses’ practice. These nurses will meet a great number of fathers and are in a position to detect a father who is suffering from PND. Therefore, the aim of this study was (a) to explore CHC nurses’ experiences of observing depression in fathers during the postnatal period; and (b) to explore the hindrances of observing these fathers.

## Methods

### Design

This descriptive qualitative study was carried out based on the reflective lifeworld research (RLR) approach developed by Dahlberg et al. [15]. This approach is based on an interest in the lifeworld and human experiences. A critical and reflective attitude was required during the research process. This approach comprises an open and sensitive attitude to lived experiences as narrated by the participants, which requires the researchers to critically reflect on their own influence on the research process and to question their preconceptions.

### Participants

In this study, a purposive sample of ten CHC nurses who specialize in pediatric care participated. The nurses were recruited for interviews in 2014 from six primary health-care settings with units for child health care in five municipalities in the western part of Sweden. Before the study was carried out, the heads of the units gave their permissions. The inclusion criteria was that the nurses should have at least one year of experience of working in child health care; all nurses except for one at these units fulfilled our criteria. Sixteen nurses were asked to participate. Of those, five declined to participate due to heavy workloads and one cancelled the interview due to sickness. Finally, ten CHC nurses participated, all of them women aged 40-59.

### Data collection

Data were collected through qualitative and semi-structured interviews [15]. The interviews focused on depression in fathers during the postnatal period as the main area for the interviews. The CHC nurses were encouraged to describe their professional experiences of observing depression in fathers in the postnatal period. They were also asked to give examples from situations when having met or observed fathers with postnatal depression. Follow-up questions were used for clarification of the descriptions by the participants, such as “Can you tell me more about that?” ”What do you mean?” and so on. The nurses were also asked to give examples from everyday practice. All interviews were audio recorded and transcribed verbatim.

### Analysis

In this study, a qualitative thematic analysis, inspired by the reflective lifeworld approach as described by Dahlberg et al. [15] was performed in order to describe CHC nurses’ experiences of observing depression in fathers in the postnatal period. The analysis started with an intensive reading of all interview material. After the reading, those parts of the data which unfolded meanings and lived experiences related to the study aim, were sought. These meaning units were identified in the interviews. The meanings were then searched through for similarities and differences in order to grasp patterns. The analysis and meanings found were discussed by the authors. The analysis involved a dialectical movement between the whole, the part, and the whole of the text. Finally, the analysis resulted in descriptions of the phenomenon and its related meanings, presented in the findings below as a main theme and five related themes. We discussed the analysis several times among ourselves, and we ventured to question our pre-understanding to minimize its negative influence on the results and the description of the themes. Our aim was to provide enough information so that the reader could evaluate the use of method and the findings that are exemplified in the quotations.

### Ethical considerations

This study follows the ethical regulations and conforms to the Declaration of Helsinki (World Medical Association 2008). According to Swedish legislation ethical approval was not needed for this study [16]. The study complies with the ethical standards for research, including respect for confidentiality. This means that the four ethical principles; respect for autonomy, beneficence, non-maleficence and justice were considered. The participants were given both oral and written information about the study. After the information written informed consent was obtained from all participants. Participants were informed that they could withdraw from the study whenever they wanted, without giving any explanation and without any effects on their work. They were guaranteed confidentiality.

## Results

Through the analysis, one main theme and five related themes were identified. The main theme was *Postnatal depression in fathers is experienced as being vague and difficult to detect*, something which was related to the following themes:

- Having limited and varied experiences of recognizing fathers with depression during the postnatal period
- Endeavoring to establish contact with the fathers
- Finding out about the father’s health status through the mother
- Lacking routines to assess the health and well-being of fathers
- Different gendered-parenting practices as a hinder for fathers’ engagement

Hindrances to the recognition of fathers with depression during the postnatal period were experienced. Even if CHC nurses were aware of the fact that fathers can suffer from depression after childbirth, difficulties were experienced in relation to this form of health problem and also with respect to the nurses’ limited contacts with most fathers. To grasp the health status of the father was hard, and experiences of fathers with such problems were limited. This can be complicated when routines are lacking or when there is no awareness of the influence of gender attitudes in daily work.

### Having limited and varied experiences of recognizing fathers with depression during the postnatal period

The CHC nurses had varied experiences of recognizing fathers with depression and they described difficulties in grasping the health status of the fathers after childbirth. Those who met fathers who were not feeling well experienced difficulties identifying such problems. It was experienced as rare to recognize fathers who had depressive symptoms. Even though the nurses were aware of health problems among fathers, they thought that it could be hard for the fathers to talk about them when not seeing them regularly. The nurses experienced it as rare that any father contacted them regarding health problems.

*In some way, it is easier to grasp the mothers: they are usually the ones to visit. Meeting the father just once makes it really hard to read and understand them, and then it’s certainly not easy for them to open up to someone they haven’t met. So, it’s difficult to identify the fathers. (4)*

Even if their experiences were limited, some of the nurses had recognized fathers as being depressed. Childbirth was experienced as stressful for the fathers, something that could aggravate their earlier problems. They said that fathers who had health problems before the birth of their child could be at risk of developing depressive symptoms after childbirth. Few fathers had come forward and contacted the nurse for consultation or talked about having depressive symptoms during a visit. A hindrance experienced was that the nurses seldom met the fathers regularly during the postnatal period, which complicated recognition of those with problems.

*They do not come. It is a difficulty that you seldom meet the fathers. (3)*

### Endeavoring to establish contact with the fathers

The CHC nurses attempted to establish contact with the fathers. During the first meeting with the newborn child and its parents, often in the family’s home, they said that they wanted the father to be at home during the visit. First-time fathers were experienced to be more eager to be present at the visit than fathers with more than one child. The nurses expressed a certain need to involve the father which seldom occurred spontaneously. The nurses have the newborn child’s best interests in focus. They mean that the well-being of the newborn child is related also to the well-being of the father. One of the nurses described the importance of asking questions to the father as one way to perceive how he feels:

*That's the most important work we have, to see and ask questions about what it like really is. It affects the child if the family is not feeling well. So it's really important to involve the fathers—they belong to the family and the child. (7)*

The nurses made efforts to establish contact with the fathers, for example by being open to their verbal and nonverbal signals and by asking questions. When unable to establish contact with the fathers, the nurses experienced the risk of not identifying their health status. In communication with the father, they expressed a need to be sensitive to what the father expressed.

*To ask questions, that’s the only thing I can do. And then I have to be very attentive and listen to what the father says. (10)*

One challenge was to relate to the father, not solely to the mother, when trying to establish contact with the family and then especially the father. In spite of this, they found it difficult to know how the father was feeling.

*Even if we ask the parents how they are feeling, we seldom learn how the fathers are doing. (10)*

### Finding out about the father’s health status through the mother

Even if it often was hard to establish contact with the father, the nurses experienced that they sometimes could grasp the health status of the father through the mother. It was not always the fathers themselves who told the nurses about how they were feeling.

*A few times I have had fathers who were obviously feeling bad. Then it always was the mothers who told me. I haven’t met any father who told me himself that he was in a bad mood and felt that it was hard. It has been the mother who has told me about his health, about the father’s not feeling well. (8)*

When the information about the health status of the father is not primarily from the fathers themselves, the information becomes ambiguous. The nurses felt uncertain about the reliability of the information from the mothers, which may allow for interpretation.

*But it is secondhand information from the other parent, not that reliable. (9)*

The mothers were described as being able to provide the nurses with important information about the fathers. Even if the information on the health status of the father was from a secondary source, which could be problematical, the nurses felt it was important to follow up on any negative signs or symptoms.

### Lacking routines to assess health and well-being of fathers

The nurses experienced that care at the CHCs was generally directed toward the child and mother, not the entire family. For example, they said that they had routines to screen for PND in mothers but not fathers.

*I often say that we focus on the mothers when using the EPDS, because it has not been developed for fathers. But we are also interested in how fathers feel. So that you open up, that you feel like it is okay to talk. But at the same time, we are more focused on the mothers. (2)*

The nurses tried to balance and compensate for this inequality by inviting the fathers to talk about their experiences. A need for tools in their daily work was described by the nurses. They were concerned about how to detect depressive symptoms and health problems in fathers.

*You would like to get one, just like the EPDS for mothers, for fathers too. One that is validated. It would be much easier for us. If we hadn’t had that for mothers, we would not have been able to identify them either. (1)*

Not only was the CHC experienced as focusing on the child and mother, not the father, the nurses also described themselves as being more involved with the mother in their daily work. They said that they would benefit from routines that helped them assess the father’s health. When the environment seems to focus on mothers, the nurses found it difficult at times to recognize depressive symptoms in fathers during the postnatal period. They were not satisfied with this situation but did not know how to change it.

*We don’t have a method for how to deal with fathers who feel bad. Today we’re doing nothing and it doesn’t feel good. We need a methodology that involves fathers. (10)*

### Different gendered-parenting practices as a hinder for fathers’ engagement

Attitudes were experienced among the nurses about what was seen as typically male or female and what to expect from female or male parents. Some gender differences were thought of as hindrances to the recognition of PND in fathers.

*We don’t know what they are like; it’s probably related to masculinity and femininity. Men have a harder time talking about their emotions and how they feel. Few dads, I can count them all on the fingers on one hand, have come here and said that they feel really bad after the delivery and that they need help and support. They just don’t do that. (10)*

The nurses reflected on their own gender attitudes as influencing their daily work. For example, if both parents arrived to the CHC, the nurses expressed that they would hear themselves say “So nice, that the father is present today,” but they would never have said the same about the mother’s presence. Another example is the home visit where they described their presumptions about the mother being present during that visit:

*I mean, if I do a home visit and the father would sit there alone with the baby, saying that “The mother is in town on an errand”, then I should think that would be really weird. But I would not react the same way if it had been the other way around. (10).*

Such attitudes were experienced on the part of both the nurses themselves and the parents they met. Fathers seemed more likely to be laidback, letting the mother be the one to take care of the child.

*It feels like some fathers automatically step back and let the mothers step forward, so that the mothers take some kind of leading role for the child. (8)*

Both verbal and nonverbal communication signaled that the mother was more important to the child than the father. Such attitudes, when reflecting on them in the interview, were experienced as hindrances to equal care and the recognition of PND in fathers.

*The dad often turns to the mom when talking; if the dad talks, he looks at her to get confirmation from her toward me. (9)*

## Discussion

The findings of this study reveal that CHC nurses had difficulty recognizing fathers with PND and that their experiences were limited. The nurses said that they were unsure about how to involve the fathers or how to assess their health and well-being. This is in accordance with Massoudi et al. [17], who found that the only thing CHC nurses could do was to ask parents about their well-being with the question “How do you feel”? That question was usually asked during the child’s first week; after that, further routines were missing. The authors state that there is a need to involve both parents in CHC care and to pay more attention to the fathers [17]. This is in line with our results, which suggest that routines are needed to encourage the participation of fathers in CHC care. The program that CHC nurses in Sweden work with does not seem to support the participation of fathers in CHC care. The nurses mean that they tried hard to establish contact with the father when meeting the family and the child for the first time. On the contrary, according to Fägerskiöld [12], fathers who were present described experiences of sometimes being overlooked in contact with the CHC nurse when the nurses turned to the mother [12]. The involvement of fathers is important not just for paternal well-being; as research has shown, it is also important to identify fathers with ill-health because the child’s development can be affected negatively as well [18, 19].

In our study, nurses described how fathers with earlier health problems can be at risk for PND. Risk factors for PND in fathers have been described in the literature; according to Ramchandani et al. [6], fathers with previous ill-health are at greater risk of developing postnatal depression. Fathers who live with depressed mothers are also at risk of developing depression [5]. This is in accordance with the findings of Ballard and Davies [20] who pointed out that a minority of fathers experience significant psychiatric morbidity and that it occurs in the most vulnerable families.

The findings reveal a need for screening tools for PND in fathers. The nurses said that they had such a tool for mothers but not for fathers. The mothers would benefit from routines to detect paternal ill-health. Depression in fathers after the birth of a child has been shown to be associated with an adverse impact on child development [8]. Thus, recognition of depression in fathers is important. Assessment of PND in fathers has been recommended, as well as assessment of risk factors [21]. A widely used tool for screening for PND in mothers is the EPDS, which has been shown to be a possible screening tool for depression in fathers as well [1]. However, the screening tool is not only a good thing. Gibson et al. [14] found that when the EPDS is used, it generates a proportion of false positives and misses a number of cases. However, most screening tools have such limitations. Even though they are helpful for indicating possible illnesses, they need to be used as an adjunct to clinical assessments when performing diagnoses [14, 17]. Routines for screening fathers are less well-developed and have not yet received the attention needed in research. According to Edmondson et al. [1], this is an area of importance which needs to be improved.

In this study, the recognition of fathers with PND seemed to be complicated and negatively influenced by different gendered-parenting practices. This contained conceptions or images with specific meanings related to gender differences. Narrow assumptions were experienced and the attitudes of nurses represented fixed images independent of the actual situation, which influenced their assessment of paternal health status and how to recognize fathers with depression. Delivery of health care is guided by the values and beliefs of professionals, who are part of the prevailing culture. However, such attitudes can be both positive and negative. According to Fägerskiöld [12], such attitudes were described as negative by fathers when perceiving that nurses at a CHC were more oriented toward the mother.

The findings of this study reveal difficulties in recognizing fathers with depression during the postnatal period as experienced by Swedish CHC nurses. Routines were lacking for the assessment of paternal health status, an area of importance which needs to be improved. However, this is probably not the case in Sweden only. No specific diagnostic tool has been developed to screen for paternal depression [22] and routines for screening fathers are less well-developed [1] and have not received the attention needed in research.

Furthermore, this study reveals that the nurses had gender attitudes on parenting influencing their daily work. To deal with such images, professionals would benefit from rethinking their attitudes in order to change behaviors influenced by it. Healthcare professionals need to create an atmosphere which helps both parents participate in CHC care. Open and flexible care free from gender attitudes may be necessary to support the well-being of the whole family.

The need to detect all signals of paternal health status in fathers suffering from PND needs to be acknowledged, maybe fathers should also be screened just like the mothers. Future studies may focus on creating and validating an instrument similar to EPDS in order to detect PND in fathers. In addition, working methods are needed to overcome gender stereotypes. To provide the best patient care, this knowledge needs to be incorporated into pediatric nurses’ practice. Care professionals at CHCs must create an atmosphere which helps parents participate in caring for a newborn child. An open, flexible attitude toward care may be necessary to support the well-being of the entire family. Pediatric nurses are central in family health-promoting care, especially in primary healthcare settings. Cornerstones of pediatric nurses’ practice are to prevent, educate, assess and refer patients to proper care when needed [22]. Overall, more attention needs to be paid to PND in fathers.

### Strengths and limitations

This qualitative study illuminates the experiences of CHC nurses in recognizing PND in fathers. In order to make it possible for the reader to agree with and understand the logic of the findings in this study, we strived to be as clear as possible when describing the research process; the participants, the data collection, and the analysis. In striving for credibility, the procedures and methods were presented as thoroughly as possible. In addition, quotations were used to show that the findings were grounded in the interview texts in order to assure conformability. Qualitative results can never be universal, but the knowledge can still be transferred to contexts other than the original one [15]. We argue that findings from this study can be useful for pediatric nurses who meet fathers and are in a position to detect a father who is suffering from PND. The outcome of the study provides awareness of hindrances to the recognition of PND in fathers. The strength of this study is that the interviews were replete with meanings and descriptions and the methodological approach was found to be suitable.

Previous research in this area is limited and more attention is needed, both in research and in clinical practice. However, this study covered a specific region of Sweden and group of nurses, which may be a limitation.

## Conclusions

The findings of this study suggest the following:

- PND was experienced as a health problem in fathers which was vague and difficult to detect
- Routines are needed to screen for PND in fathers in order to identify those who are at risk of developing PND
- Professionals at CHCs would benefit from changing their working methods in order to overcome gender attitudes influencing their encounter with parents
- An open, sensitive attitude is needed to all kinds of signals of paternal health status in order to identify fathers with depressive symptoms.
